# RTA404, an Activator of Nrf2, Activates the Checkpoint Kinases and Induces Apoptosis through Intrinsic Apoptotic Pathway in Malignant Glioma

**DOI:** 10.3390/jcm10214805

**Published:** 2021-10-20

**Authors:** Tai-Hsin Tsai, Ann-Shung Lieu, Tzuu-Yuan Huang, Aij-Lie Kwan, Chih-Lung Lin, Yi-Chiang Hsu

**Affiliations:** 1 Division of Neurosurgery, Department of Surgery, Kaohsiung Medical University Hospital, Kaohsiung 807, Taiwan; teishin8@hotmail.com (T.-H.T.); annshunglieu910077@gmail.com (A.-S.L.); peigen0112@gmail.com (A.-L.K.); linzhilong800134@gmail.com (C.-L.L.); 2 Department of Surgery, School of Medicine, College of Medicine, Kaohsiung Medical University, Kaohsiung 807, Taiwan; 3 Graduate Institutes of Medicine, College of Medicine, Kaohsiung Medical University, Kaohsiung 807, Taiwan; 4 Department of Neurosurgery, Changhua Christian Hospital, Changhua 500, Taiwan; zhihuizhang53@gmail.com; 5 School of Medicine, I-Shou University, Kaohsiung 824, Taiwan

**Keywords:** RTA404, malignant glioma, apoptosis, checkpoint kinase

## Abstract

**Background:** Malignant glioma (MG) is an aggressive malignant brain tumor. Despite advances in multidisciplinary treatment, overall survival rates remain low. A trifluoroethyl amide derivative of 2-cyano-3-,12-dioxoolean-1,9-dien-28-oic acid (CDDO), CDDO–trifluoroethyl amide (CDDO–TFEA) is a nuclear erythroid 2-related factor 2/antioxidant response element pathway activator. RTA404 is used to inhibit proliferation and induce apoptosis in cancer cells. However, its effect on tumorigenesis in glioma is unclear. **Methods:** This in vitro study evaluated the effects of RTA404 on MG cells. We treated U87MG cell lines with RTA404 and performed assessments of apoptosis and cell cycle distributions. DNA content and apoptosis induction were subjected to flow cytometry analysis. The mitotic index was assessed based on MPM-2 expression. Protein expression was analyzed through Western blotting. **Results:** RTA404 significantly inhibited the cell viability and induced cell apoptosis on the U87MG cell line. The Annexin-FITC/PI assay revealed significant changes in the percentage of apoptotic cells. Treatment with RTA404 led to a significant reduction in the U87MG cells’ mitochondrial membrane potential. A significant rise in the percentage of caspase-3 activity was detected in the treated cells. In addition, these results suggest that cells pass the G2 checkpoint without cell cycle arrest by RTA404 treatment in the MPM-2 staining. An analysis of CHK1, CHK2, and p-CHK2 expression suggested that the DNA damage checkpoint system seems also to be activated by RTA404 treatment in established U87MG cells. Therefore, RTA404 may not only activate the DNA damage checkpoint system, it may also exert apoptosis in established U87MG cells. **Conclusions:** RTA404 inhibits the cell viability of gliomas and induces cancer cell apoptosis through intrinsic apoptotic pathway in Malignant glioma. In addition, the DNA damage checkpoint system seems also to be activated by RTA404. Taken together, RTA404 activated the DNA damage checkpoint system and induced apoptosis through intrinsic apoptotic pathways in established U87MG cells.

## 1. Introduction

Glioma, the most common type of brain tumor, is classified by the World Health Organization (WHO) into four grades based on histologic features [1,2]. There are four grades of glioma, and each has different types of cells present and different treatment strategies. A grade III glioma, or malignant glioma (MG), is a tumor of the anaplastic cells. These tumors are very aggressive. WHO grade IV, also called glioblastoma multiforme (GBM), is angiogenic and can cause necrosis. Glioblastoma multiforme are the most aggressive tumors of the central nervous system [3]. Despite advances in multi-disciplinary treatment, MG and GBM continue to have poor overall survival rates. With standard treatment, median survival for adults with an anaplastic astrocytoma is about two to three years [4]. For adults with the more aggressive glioblastoma, treated with concurrent temozolomide and radiation therapy, median survival is about 14.6 months [5]. Therefore, identifying new and effective anticancer drugs and understanding their mechanism of action in treating malignant tumors is essential.

The induction of apoptosis and the arrest of the cell cycle are two of the best approaches for suppressing cancerous tumors [6]. Apoptosis is a form of programmed cell death that results in the orderly and efficient removal of damaged cells, such as those resulting from DNA damage or during development [7,8]. Apoptosis deregulation is associated with a wide variety of human diseases [8], and apoptosis deregulation is also a hallmark of cancer [9]. Most anticancer drugs currently used in clinical settings develop the intact apoptotic signaling pathways to trigger cancer cell death [10]. Therefore, a better understanding of the apoptotic signaling pathways may improve the efficacy of cancer therapy. CDDO-trifluoroethyl amide (CDDO-TFEA, RTA404) has the effect of inducing apoptosis and inhibiting the growth of a variety of tumor cells at high doses. RTA404 is a new anti-cancer drug with great potential.

A component of Chinese herbal medicine prescribed for hepatitis, oleanolic acid has been chemically modified to create an oleanane triterpenoid, 2-cyano-3-,12-dioxoolean-1,9-dien-28-oic acid (CDDO) [11]. The therapeutic effect of CDDO stems from its ability to upregulate nuclear erythroid 2-related factor 2 (Nrf2) by changing the conformation of the Nrf2-repressing Kelch-like erythroid cell-derived protein with cap’n’collar homology associated protein 1 (Keap1) [12]. CDDO–trifluoroethyl amide (RTA404), a trifluoroethylamide derivative of CDDO, has antioxidation, anti-inflammatory, and antiproliferative effects, in addition to an enhanced ability to cross the blood–brain barrier [13]. Currently, CDDO–TEFA has been used to treat neurodegenerative diseases, neuroinflammatory diseases, and neuro oncology cancers. It also enhances Nrf2 expression and signaling in various models of neurodegeneration [14], including those that simulate multiple sclerosis [15], amyotrophic lateral sclerosis [16], and Huntington’s disease [17]. CDDO–TEFA has exerted neuroprotective effects in some animal models of degenerative conditions such as ischemic stroke [13] and autoimmune encephalomyelitis [15]. Moreover, in one study, it induced apoptosis in neuroblastoma cells [18]. In addition, in GMC cells, RTA404 can inhibit proliferation, cell locomotion, and cell cycle progression and induce apoptosis [19].

The present study focuses on RTA404 and its chemopreventive activities against glioma cells, specifically U87MG cells, which are malignant glioma cells found in the human brain. We examined RTA404′s effects on cell growth and cell cycle regulation and evaluated the expression levels of downstream molecules. There is strong evidence that RTA404 inhibits the proliferation of gliomas and induces cancer cell apoptosis. However, we cannot determine the effect of RTA404 on the cell cycle of glioma. The novelty of this study is that RTA404, a novel and potential anticancer drug, induces cell apoptosis through an intrinsic apoptotic pathway in established U87MG cells.

## 2. Materials and Methods

### 2.1. Materials 

2-cyano-3,12-dioxo-N-(2,2,2-trifluoroethyl)-oleana-1,9(11)-dien-28-amide, CDDO-TFEA (RTA404), was purchased from Cayman Chemical, DMSO (dimethyl sulfoxide) and PrestoBlue™ Cell Viability Reagent (PB) were purchased from ThermoFisher (ThermoFisher Scientific 168 Third Avenue Waltham, MA, USA). Cell culture medium (DMEM), fetal bovine serum, antibiotics, sodium pyruvate, trypsin, and phosphate-buffered saline (PBS) were obtained from Gibco, BRL (Grand Island, NY, USA), and polyvinylidene fluoride membrane (PVDF) (Millipore), and molecular weight markers from Bio-Rad (BioTek Instruments, Headquartered in Winooski, VT, USA). All other reagents and compounds were of analytical grade.

### 2.2. Cell Culture

Human brain malignant glioma U87MG cells were obtained from Bioresource Collection and Research Center (BCRC, Hsinchu, Taiwan). All cell lines were incubated in an atmosphere containing 5% CO_2_ at 37 °C. U87MG cells were cultured in an RPMI1640 medium with supplemental 10% fetal bovine serum (FBS) and U87 MG cells in modified Eagle’s medium (MEM) with supplemental 10% FBS.

### 2.3. Cell Viability

A density of 5000 cells was suspended in a culture medium containing 10% FBS and placed in a 96-well plate (0.1 mL of medium per each well) and incubated in an atmosphere containing 5% CO_2_, saturated humidity, and 37 °C for 24 h. The cells were added with 0, 1, 1.5, 2 μM RTA404 and incubated with PrestoBlue™ Cell Viability Reagent for 10 min. The reaction was measured at 570 nm using a multiwell plate reader (lQUANT; BioTek Instruments, Inc., CA, USA). When there were no cells, we subtracted the background absorbance of the medium. All samples were assayed at least in triplicate, and the mean was calculated for each experiment. Results were expressed as a percent of control, control being 100%. Each experiment was performed in triplicate with a mean (+/−SEM) used to express results.

### 2.4. Cell Cycle Analysis

The U87MG cells were plated in 6-well plates (1 × 10^6^) in a cell incubator and cultured overnight. The next day, the cells were centrifuged in a 10 mL centrifuge tube and the supernatant was collected. They were washed two times in PBS and added with 1× Trypsin. They were then placed in a 37 °C oven for 1–2 min. Once the cells fell off, they were collected in a centrifuge tube run at 2500 rpm for 5 min to rjapanemove the supernatant. Then, 1 mL of PBS was added to wash the remaining culture solution and the cells were centrifuged again at 2500 rpm for five minutes. 500 µL of PBS was added to break up the cell pallet and then 500 µL of 70% ethanol was slowly added to the cells for fixation. They were placed in a refrigerator and left there overnight. The next day those cells were centrifuged at 2500 rpm for 5 min, and the supernatant containing Ethanol was removed. The cells were washed in one ml PBS. 5 μL of RNAse A 100 mg /mL was added to PBS and placed in an oven at 37 °C. 

To facilitate cell cycle analysis, a fluorescent nucleic acid dye PI was used to identify the proportion of cells in each of the three inter-phase stages. After a 30-min reaction time, 20 µL of propidium iodide 2 mg/mL (final concentration 40 µg/mL) was added and the cells were placed in an oven at 37 °C for 15 min. The cells were treated with RTA404 for 24 h followed by harvesting and fixing in 1 mL of ice-cold ethanol (70%) at −20° C for at least 8 h. DNA was stained with PI/RNaseA staining buffer, and the cell cycle was analyzed using a FACSCalibur flow cytometer. Data were interpreted using WinMDI 2.9 software.

### 2.5. Apoptosis Measurement

The cells were cultured in 6 well culture plates (Orange Scientific, Belgium, EU). After exposure to RTA404 for 4 h, the cells were harvested by centrifugation, resuspended in, and incubated with 1 × annexin-binding buffer containing 5 lL of annexin V-FITC and 1 lL of propidium iodide (PI) (100 mg/mL), and incubated at room temperature for 15 min. The stained cells were analyzed on a FACSCalibur flow cytometer (BD Pharmingen, CA, USA) using WinMDI 2.9 free software (BD Pharmingen, CA, USA).

### 2.6. Evaluation of Mitochondrial Membrane Potential

The cells were seeded into 24-well plates (Orange, UK). Following treatment with RTA404 for 6 h, we added 10 µg/mL JC-1 (Sigma, Ron konkoma, NY, USA) to the culture medium at 50 µL/well, which was then incubated at 37 °C for 20 min for mitochondrial staining. After being washed twice with warm PBS, the cells were fixed with 2% paraformaldehyde and detected by FACS Calibur flow cytometer (JC-1). Data were analyzed using WinMDI 2.9. JC-1 was also detected by using fluorescence microscopy (Olympus CKX41 and U-RFLT 50, Tokyo, Japan).

### 2.7. Western Blotting

All samples were lysed in 200 μL of lysis buffer. A total of 50–75 μg of protein per sample was loaded onto 10–12% sodium dodecyl sulfate-polyacrylamide gel electrophoresis membranes for electrophoretic separation and then transferred to PVDF membranes and subjected to electrophoresis at 50 V for 4 h. After blocking overnight with Odyssey blocking buffer (USA), the membranes were incubated with primary antibodies [Cyclin A2 (1:1000; proteintech; 18202-1-AP), NRF2 (1:1000; proteintech; 16396-1-AP), CHK2 (1:1000; abgent.com; AP4999a), p-CHk2 (1:1000; abgent; AP50241), CHK1 (1:1000; proteintech; 22018-1-AP), p21 (1:1000; Cell Signaling; #2947), and β-actin (1:20,000; Sigma; A5441)] for 2 h at room temperature. Subsequently, the membranes were washed several times and then incubated with a corresponding secondary antibody (IRDye Li-COR, USA) at a dilution of 1:20,000 for 30–45 min. Antigens were then visualized using a near-infrared fluorescence imaging system (Odyssey LICOR, NE, USA), and the data were interpreted using the Odyssey 2.1 software or a chemiluminescence detection kit (ECL; Amersham Corp., Arlington Heights, IL, USA).

### 2.8. Mitotic Index Analysis 

The mitotic index was assessed based on MPM-2 (anti-phospho-Ser/Thr-Pro) expression. After treatment with CDDOTFEA, cells were harvested and fixed in 70% ethanol overnight. The cells were then washed and suspended in 100 µL of IFA-Tx buffer (4% FCS, 150 nM NaCl, 10 nM HEPES, 0.1% sodiumazide, and 0.1% Triton X-100) with the MPM-2 antibody at room temperature for 1 h. The cells were then washed and resuspended in IFA-Tx buffer with a rabbit anti-mouse FITC-conjugated secondary antibody (1:50 dilution; Serotec) for 1 h at room temperature in the dark. Finally, the cells were washed and resuspended in 500 µL of PBS with 20 µg/mL PI (Sigma) for 30 min in the dark. MPM-2 expression was measured by a FACSCalibur flow cytometer. The data were analyzed using WinMDI 2.9.

### 2.9. Data Analysis

The data are expressed as the mean ± standard error of the mean of at least three independent experiments. Student’s *t*-test or one-way analysis of variance with Scheffe’s posthoc test was used for statistical analysis. A *p* value of <0.05 was considered statistically significant.

## 3. Result

### 3.1. RTA404 Reduces the Viability of U87MG Cells by Both Time and Does Dependent Manner

**Figure 1:**
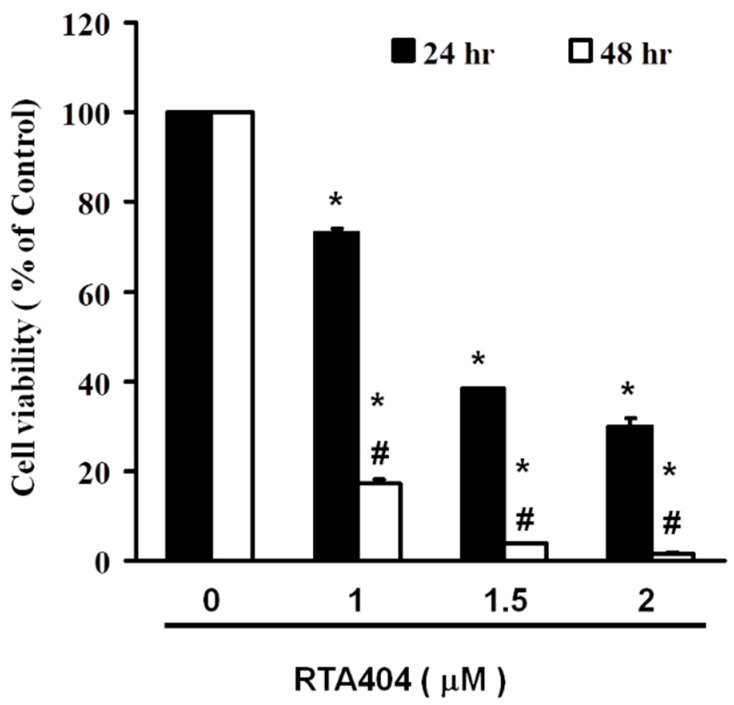
Viability of U87MG cells after 24–48 h of RTA404 treatment. RTA404 mediated the survival of U87MG cells by inhibiting proliferation. Cells were treated with increasing doses of RTA404 for 24–48 h in vitro. The survival rate of the treated cancer cells was measured using a PB assay. Results are expressed as percentages of the control, with the control being 100%. All data are reported as means ± standard errors of the mean on the basis of the results of at least three separate experiments. Statistical analysis was performed using the *t* test, with differences between the treatment and control groups (# vs. 24-h treatment) considered significant at *p* < 0.05, as denoted by * and # standard errors of the mean.

To determine whether RTA404 mediates the cell viability of U87MG cells, we have tested the concentration range from 100 microM to 390 nM in the initial experiment (24 h treatment), follow-up experiments were taken with increasing doses of RTA404 (0, 1, 1.5, and 2 μM) for 24 h and then cell viability was measured by using peripheral blood (PB). We used normal lung fibroblasts MRC-5 cells as a control group for viability. There was no change in normal lung fibroblasts MRC-5 cells (Normal lung fibroblasts; data not shown). As shown in Figure 1, the cell viability of the U87MG cells decreased significantly inversely with the number of RTA404 doses (24 h: y = −24.541x + 121.65, R^2^ = 0.9544; 48 h: y = −30.847x + 107.82, R^2^ = 0.7268) administered over 24–48 h.

### 3.2. RTA404 Induced Apoptosis in U87MG Cells by Does Dependent Manner

**Figure 2:**
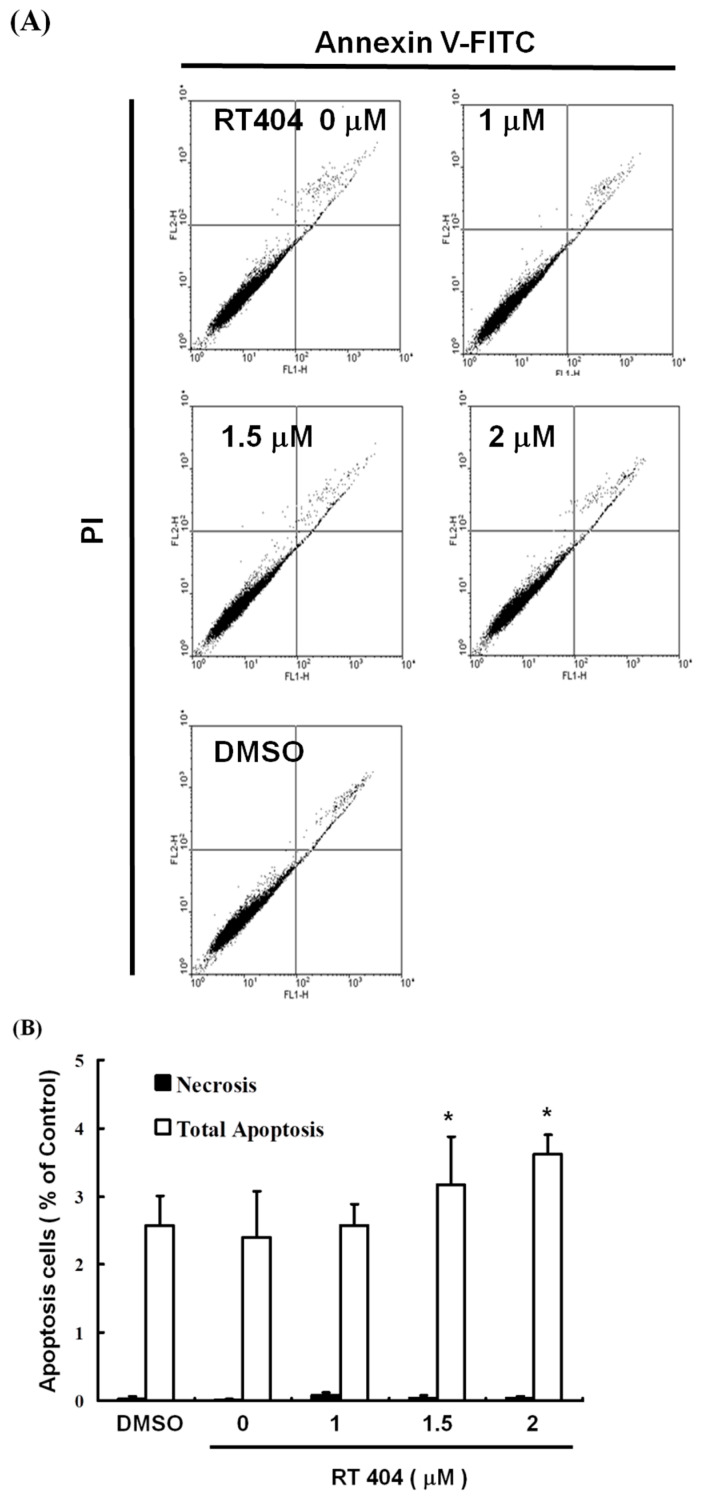
RTA404 induced apoptosis in U87MG cells. (**A**) Annexin V-FITC and (**B**) PI staining. Results are expressed as a percentage of the control, with the control being 100%. All data are reported as means ± standard errors of the mean on the basis of the results of at least three separate experiments. Statistical analysis was performed using the *t* test, with differences between the treatment and control groups considered significant at *p* < 0.05, as denoted by *.

To elucidate the role of RTA404 in the apoptosis of U87MG cells, we treated the cells with RTA404 (0, 1, 1.5, and 2 μM) for 4 h. This was followed by Annexin V-FITC detection and propidium iodide (PI) staining. Cell populations and apoptotic ratios were analyzed through flow cytometry. The Annexin-FITC/PI assay revealed significant changes in the percentage of apoptotic cells under and not under RTA404 treatment (i.e., the control cells; Figure 2A). The percentage of apoptotic cells increased significantly in the treated cells relative to in the untreated cells (Figure 2B; y = 0.4287x + 0.18667; R^2^ = 0.963).

### 3.3. RTA404 Treatment Causes the Loss of Mitochondrial Membrane Potential

**Figure 3:**
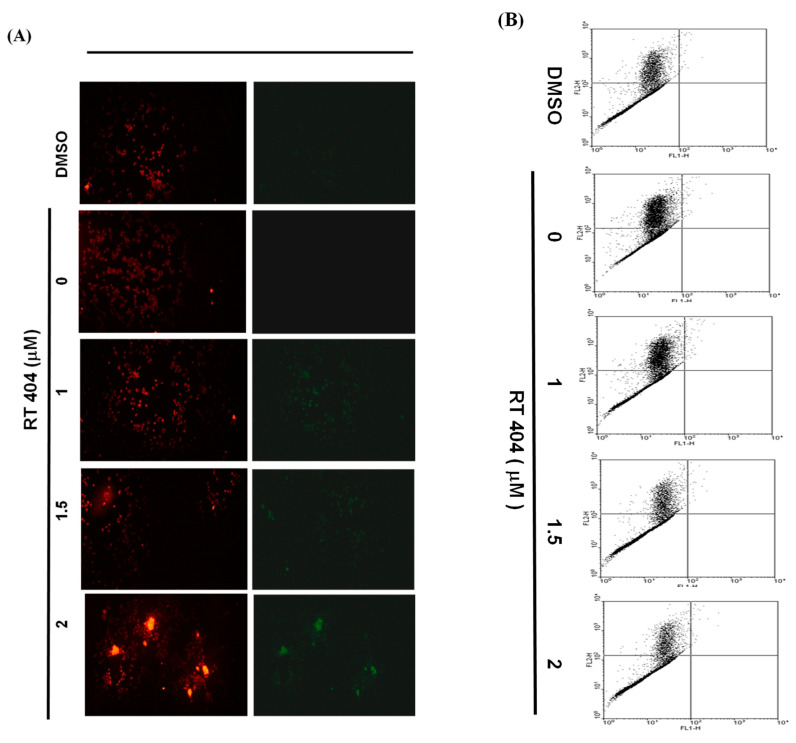

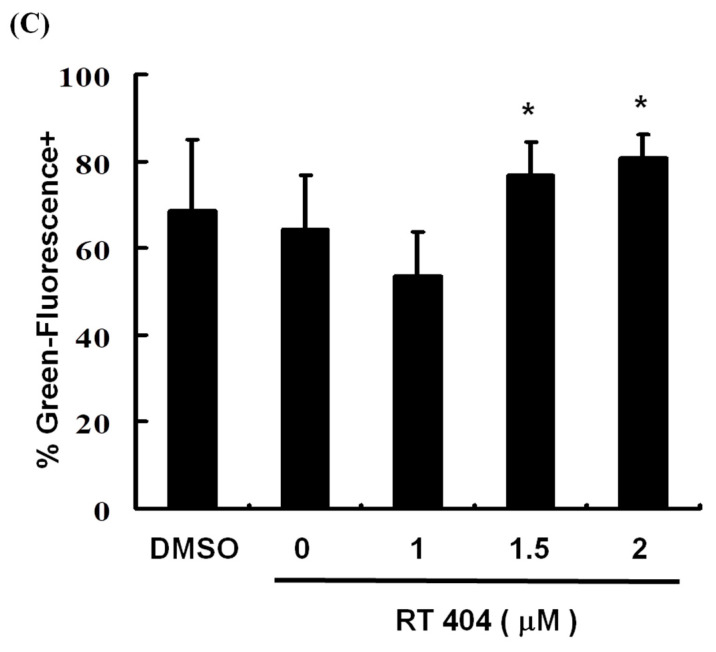
Disruption of the mitochondrial membrane potential caused by RTA404 treatment. JC-1 was also detected by using fluorescence microscopy (**A**) and flow cytometry (**B**). (**C**) Results are expressed as a percentage of the control, with the control being 100%. All data are reported as the means ± standard errors of the mean on the basis of the results of at least three separate experiments. Statistical analysis was performed using the *t* test, with differences between the treatment and control groups considered significant at *p* < 0.05, as denoted by *.

To elucidate the role of RTA404 in the apoptosis of U87MG cells, we treated the cells with RTA404 (0, 1, 1.5, and 2 μM) for 4 h. This was followed by a JC-1 assay. The loss of mitochondrial membrane potential (ΔΨm), an early cellular metabolism event coinciding with caspase activation, is a hallmark of apoptosis. In nonapoptotic cells, JC-1 is present as a green monomer in the cytosol, but it accumulates as red aggregates in the mitochondria. In apoptotic and necrotic cells, although JC-1 is present in a monomeric form, it stains the cytosol green. To explore the possible effect of RTA404 on ΔΨm in the U87MG cells, we used JC-1 as a dye to assess ΔΨm loss in the treated cancer cells. As shown in the left panel of Figure 3A, the cells’ ΔΨm was reduced after treatment with RTA404. The right panel of Figure 3A presents typical FL-1/FL-2 dot plots for apoptotic and nonapoptotic U87MG cells stained with JC-1. The untreated cancer cells did not experience apoptosis, resulting in red fluorescing JC-1 aggregates. The green fluorescing monomers in the lower part of the figure indicate apoptotic cells. In sum, treatment with RTA404 led to a significant reduction in the U87MG cells’ ΔΨm (Figure 3B, y = 7.2465x + 50.738; R^2^ = 0.5709). The results displayed in Figure 2 and Figure 3 suggest that RTA404 mediates the survival of the U87MG cell line. Thus, we postulated that the proliferation of these cells was inhibited by apoptosis pathways.

### 3.4. RTA404 Treatment Increases the Numbers of Active Caspase-3

**Figure 4:**
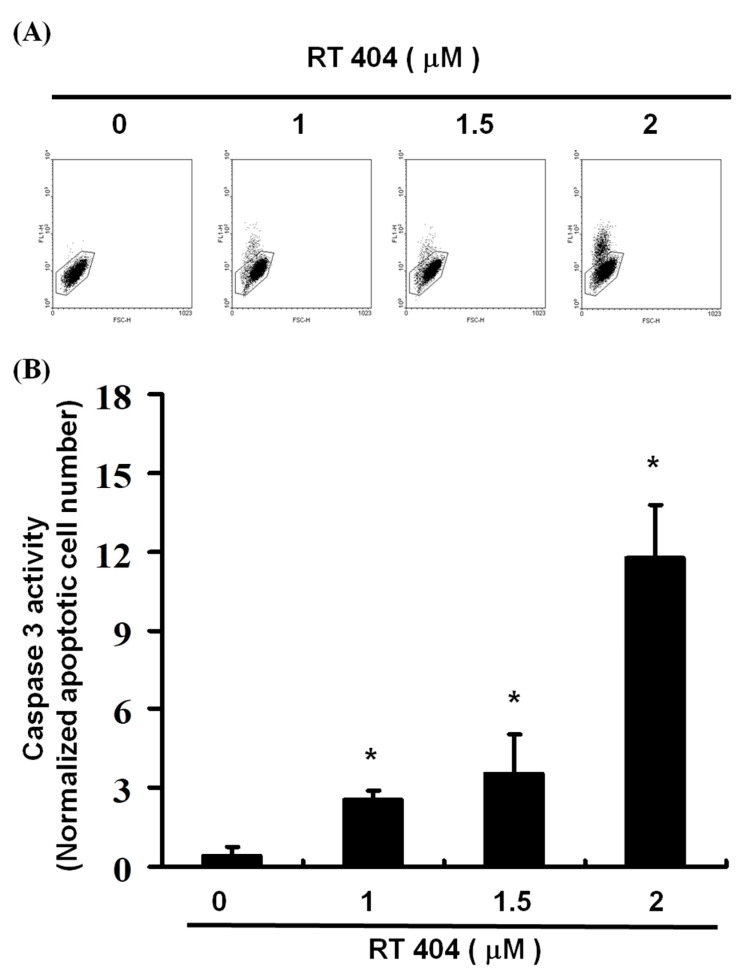
(**A**) Numbers of active caspase-3 induced by RTA404 treatment on the U87MG cell line. (**B**) Results are expressed as a percentage of the control, with the control being 100%. All data are reported as the means and standard errors of the mean on the basis of the results of at least three separate experiments. Statistical analysis was performed using the *t* test, with differences between the treatment and control groups considered significant at *p* < 0.05, as denoted by *.

To elucidate the role of RTA404 (0, 1, 1.5, and 2 μM) in the apoptosis of U87MG cells, we treated the cells with RTA404 for 4 h. This was followed by the caspase-3 assay and the number of active caspase 3 cells was measured. We used different concentrations (0, 1, 1.5, 2 μM) of CDDOTFEA to treat U87MG cells, and used flow cytometry to measure the number of active caspase 3 cells, and found that as the concentration of CDDOTFEA increased, the number of active caspase 3 cells increased linearly. As Figure 4 show, a significant rise in the percentage of caspase-3 activity was detected in RTA404-treated cancer cells (Figure 4A,B, y = 3.4973x − 4.1967 R^2^ = 0.8286).

### 3.5. Cell Pass the G2 Checkpoint without Cell Cycle Arrest by RTA404 Treatment in U87MG Cells

**Figure 5:**
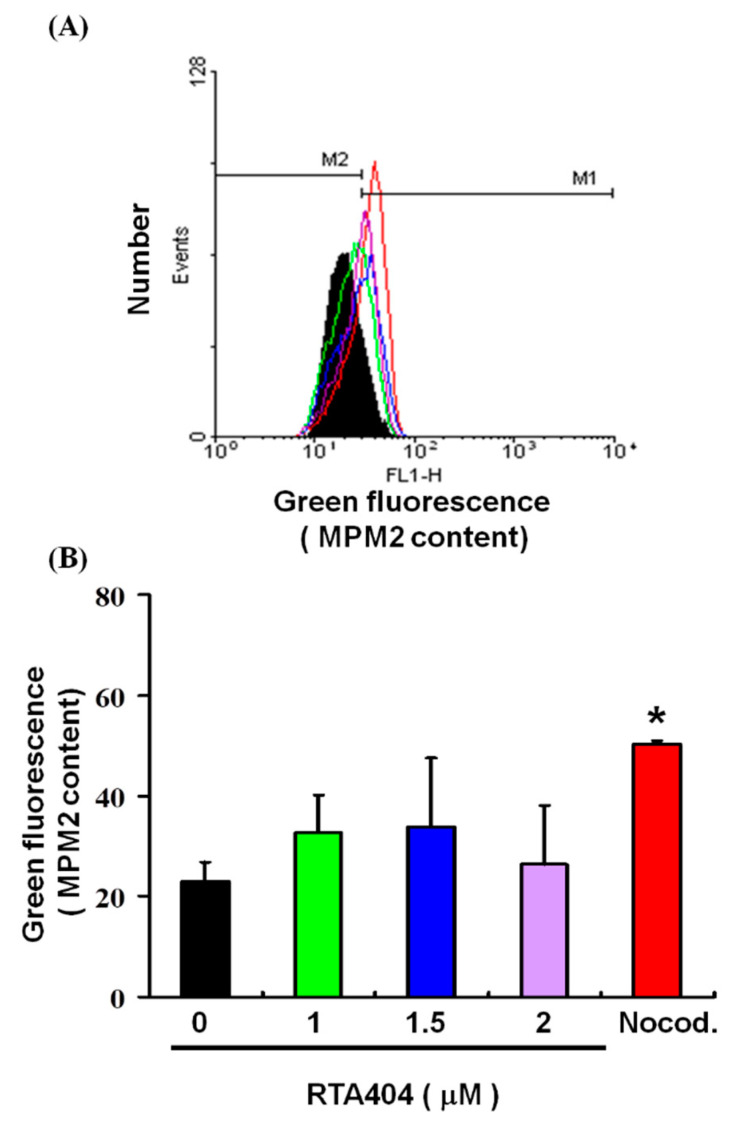
Mitotic index was assessed based on MPM-2 expression. Flow cytometry analysis of MPM-2 expression in the treated cells was conducted. (**A**) Cells were either treated or not treated with 0, 1, 1.5, or 2 μM RTA404. After 24 h of treatment, cells were fixed with 70% ethanol, stained with MPM-2 and PI, and analyzed using FACScan software. (**B**) Results are expressed as a percentage of the control, with the control being 100%. All data are reported as means ± standard errors of the mean on the basis of the results of at least three separate experiments. Statistical analysis was performed using the *t* test, with differences between the treatment and control groups considered significant at *p* < 0.05, as denoted by *.

We explored the potential role of RTA404 treatment in mitosis in the U87MG cells. The mitotic index was assessed based on MPM-2 expression. Figure 5 displays the number of cells exposed to RTA404 at various concentrations (0, 1, 1.5, and 2 μM). Figure 5A,B demonstrate that exposure to RTA404 increased levels of protein synthesis during mitosis (y = 1.194x + 25.947; R^2^ = 0.0893). There was no significant difference in comparison with the control group. These results suggest that regarding increased protein synthesis during mitosis in the MPM-2 staining, itis implied that cells pass the G2 checkpoint without cell cycle arrest in U87MG cells.

### 3.6. Protein of NRF2, p-CHK2, CHK1/2, p21 to Be Up-Regulated Expressions in RTA404-Treated U87MG Cells

**Figure 6:**
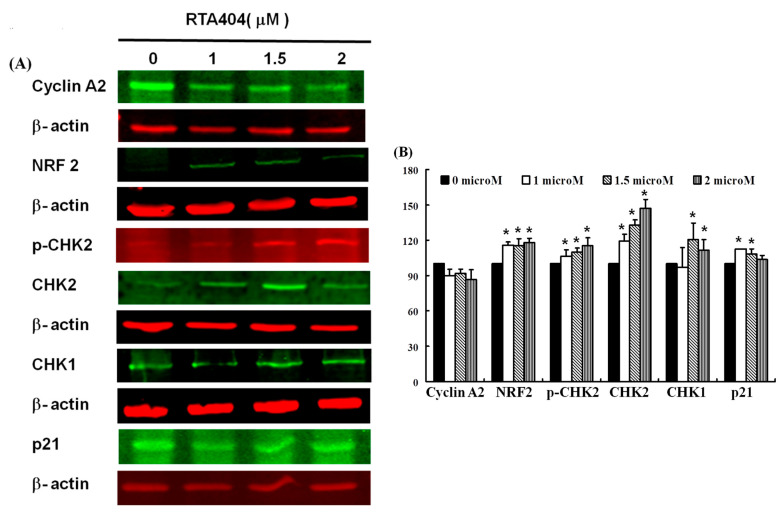
(**A**) RTA404 regulated DNA damage checkpoint kinase expression in CHK1, CHK2, and p-CHK2, as well as cyclin A2, NRF2 and p21 gene expression in the U87MG cells. Cells were treated with RTA404 (0, 1, 1.5, and 2 μM) for 24 h. Gene and protein expression was subsequently detected through Western blotting. (**B**) Results are expressed as a percentage of the control, with the control being 100%. All data are reported as means ± standard errors of the mean on the basis of the results of at least three separate experiments. Statistical analysis was performed using the *t* test, with differences between the treatment and control groups considered significant at *p* < 0.05, as denoted by *.

As shown above, we found that RTA404 induced apoptosis and inhibited *proliferation* in the U87MG cells. Figure 6A,B shows the results of our Western blot analysis of cellular proteins extracted from the brain cancer cell lines treated with RTA404 (0, 1, 1.5, and 2 μM). We measured the relative intensities of cyclin A2, NRF2, p-CHK2, CHK1/2 and p21 expression. In this experiment, we measured the relative intensities of cell cycle regulators such as cyclin A2’s expression. As Figure 6 shows, the relative intensity of cyclin A2 was not significantly different compare with the control group after RTA404 treatment. In addition, we also measured the relative intensities of human checkpoint kinases such as CHK1, CHK2, and p-CHK2 expression. The relative intensities of CHK1, CHK2 and p-CHK2 were significantly upregulated in RTA404-treated U87MG cells. Furthermore, we measured the relative intensities of the cyclin-dependent kinase 1 inhibitor, p21 expression. The intensity of p21 was significantly upregulated in RTA404-treated U87MG cells. 

## 4. Discussion 

Treatment with RTA404 reduces the cell viability of U87MG Cells by both time and dose dependent manner. RTA404 treated cells had a significant increase in the percentage of apoptotic cells, loss of mitochondria membranes potential; and rise in the percentage of caspase-3 activity. Therefore, these results indicated that incubation with RTA404 inhibited cell viability and induced cell apoptosis through the intrinsic apoptotic pathway. Although treatment with RTA404 led to cell cycle arrests in previous studies [19], without significant changes in the mitosis index we have found in RTA404 treatment. Based on the above results, it seems to imply that RTA404 activated the DNA damage checkpoint system and induced apoptosis through intrinsic apoptotic pathways in established U87MG cells.

### 4.1. RTA404 Induced Apoptosis in Malignant Glioma 

Mounting evidence indicates that apoptosis is closely related to the survival of cancer and it has emerged as a key target for the discovery and development of novel anticancer drugs [20,21,22]. The efficiency of an anticancer drug depends on the successful induction of apoptosis. The understanding of apoptosis signaling pathways and dysregulation in cancer treatment has made great progress. These advances provide new molecular targets for pro-apoptotic cancer therapies that have recently been used in drug development [21]. Based on the results of this experiment, RTA404 does induce cell apoptosis. First, RTA404 induced apoptosis in U87MG cells, and no change occurred in MRC-5 cells (normal lung fibroblasts; data not shown). Second, RTA404 reduced the cell viability of U87MG cells by inducing apoptosis, as demonstrated by the dose-dependent increase in the number of Annexin V–positive cells and the observation of nuclear chromatin condensation and DNA fragmentation therein following incubation with RTA404 (Figure 2). 

Moreover, a significant rise in the proportion of caspase-3 activity was detected in the treated cells (Figure 4). Finally, the loss of mitochondrial membrane potential, an early cellular metabolism event coinciding with caspase activation, is a hallmark of apoptosis. As revealed through fluorescent microscopy, RTA404 also reduced the aggregate-to-monomer ratio of JC-1, corresponding to the reduced number of cells, with no loss in ΔΨm (Figure 3). Taken together, RTA404 has the effect of inducing apoptosis and inhibiting the cell viability at high doses. Therefore, RTA404 is a novel and potential anti-cancer drug.

### 4.2. RTA404 Induced Apoptosis through Intrinsic Apoptotic Pathway

Apoptosis is a process of programmed cell death that is involved in intrinsic and extrinsic pathways that activate the caspase family of cysteine proteases [23,24]. The intrinsic apoptotic pathway is mediated by intracellular signals that converge at the mitochondrial level in response to different stress conditions [25,26]. Apoptotic markers such as BAX induce apoptosis through ΔΨm alteration that results in the translocation of cytochrome c from the mitochondrial inner membrane to the cytosol that triggers the signaling of caspase cascades [27,28,29]. Cytochrome c activates and causes cleaving in caspase-9, which in turn activates caspase-3 [27,30]. Caspase-9 activates downstream caspase-3 [27,30], a well-known apoptotic mediator that drives DNA damage and, subsequently, apoptosis. In the extrinsic pathway, stimulation of the TNF family of receptors results in the activation of caspase-8 [31,32], which can directly activate caspase-3 [33,34] Activation of the same target apoptotic molecules such as caspase-3 occurs [33,35]. Subsequently, the execution phase of apoptosis is initiated by the cleavage of Caspase-3 and results in cell shrinkage, chromosomal condensation, and nuclear and chromosomal DNA fragmentation [8]. 

In this study, treatment with RTA404 led to a significant reduction in the U87MG cells’ mitochondrial membrane potential (ΔΨm). As we know, the loss of ΔΨm, an early cellular metabolism event coinciding with caspase activation, is a hallmark of apoptosis. In addition, we used flow cytometry to measure the number of active caspase 3 cells, and found that as the concentration of CDDOTFEA increased, the number of active caspase 3 cells increased linearly. Base on the results, treatment with RTA404 induces the loss of glioma cell mitochondrial membrane potential that is involved in intrinsic pathways and activates caspase 3 to initiate the execution phase of apoptosis, which leads to cell death.

### 4.3. RTA404 Initiate Apoptosis by Activating DNA Damage Checkpoint System

**Figure 7:**
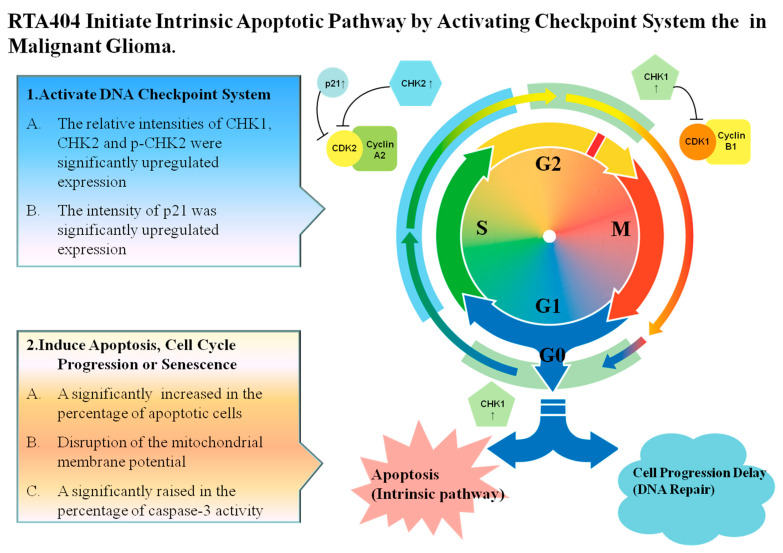
RTA404 induces apoptosis and cell cycle arrest in malignant glioma. RTA404, an activator of Nrf2, activates the checkpoint kinases and induces apoptosis through intrinsic apoptotic pathway in malignant glioma. **G1 phase:** Growth 1 phase. S phase: Synthesis phase. G2 phase: Growth 2 phase. M phase: mitosis. CDK1: Cyclin Depandant Kinase 1. CDK2: Cyclin Depandant Kinase 2. CHK1: Checkpoint Kinase 1. CHK2: Checkpoint Kinase 2.

Cell cycle deregulation, a feature unique to human cancer, involves the mutation of cell cycle–regulated genes, which allows a cell to circumvent checkpoint control systems [36]. The activated cell cycle checkpoints delay cell cycle progress to promote DNA repair. The checkpoints can also eliminate harmful damaged cells by inducing cell death, thereby protecting the organism from cancer [37]. Checkpoint kinases 1 and 2 (CHK1 and CHK2) play important signal transducer roles in cell cycle checkpoints. The task of CHK1 and CHK2 are to transmit checkpoint signals from the checkpoint kinases of the phosphatidylinositol 3 kinase family such ATM and ATR [38,39,40], which phosphorylate and activate CHK1 and CHK2. CHK2 is a stable protein expressed throughout the cell cycle. In contrast, the unstable CHK1 protein is mainly limited to the S and G2 phases [41]. According to different conditions, different pathways are involved; the ATM-CHK2-p53 pathway controls the G1 checkpoint or the ATR-CHK1-Wee1 pathway controls the S and G2/M checkpoints [42,43,44]. Alterations of CHK1 and CHK2 expression are suggested contributors to the development of both hereditary and sporadic human cancer [45]. In this experiment, we measured the relative intensities of human checkpoint kinases such as CHK1, CHK2 and p-CHK2 expression. The relative intensities of CHK1, CHK2 and p-CHK2 (Figure 7(2C)) were significantly upregulated expression in RTA404-treated U87MG cells. Based on the above results, it is implied that RTA404 activates the checkpoint kinases such as CHK1, CHK2 and p-CHK2, thereby inhibiting the CDK, causing cells to fail to cell progression, and ultimately leading to cell cycle arrest or inducing cell death. However, the little evidence in this experiment cannot determine whether RTA404 acts directly on checkpoint kinases, or RTA404 acts on upstream regulators such as ATM and ATR, or RTA404 acts on more upstream regulators such as P53 and Wee1, etc.

### 4.4. RTA404, an Activator of Nrf2, Has Multi-Pharmacological Functions

CDDO derivates belong to Nrf2activator, which can achieve anti-oxidant and anti-inflammatory cytoprotection through the Keap1-Nrf2 pathway (Kansanen et al., 2013). The relative intensities of Nrf2 were significantly upregulated in RTA404-treated U87MG cells in this experiment. However, it was found that the membrane potential of the mitochondria was destroyed, which caused caspase 3 to activate the downstream caspase cascades and lead to apoptosis. This happened because different concentrations of CDDO derivates induce different effects. At low concentrations, these synthetic triterpenoids interact with kelch-like ECH-associated protein 1 (Keap1) and activate a cytoprotective pathway, while at higher concentrations other proteins (such as JAKs, USP7, IKK, and Lonp1) are targeted to inhibit proliferation or induce apoptosis (Borella et al., 2019).

Furthermore, CDDO derivatives have multi-pharmacological functions. In previous studies, CDDO derivates can regulate the signal transmission of Keap1-Nrf2, NFKB, and the STATs pathway, and then achieve anti-oxidation, anti-inflammatory and even anti-cancer effects, and the interaction between the signal pathways are quite close and complicated (Saha et al., 2020, Krajka-Kuźniak and Baer-Dubowska, 2021). The interplay among these pathways occurs through a range of complex molecular interactions. Despite convincing evidence for functional interactions among the pathways, many aspects of the dynamic nature of cross-talk remain unknown. Because of many important aspects of co-regulation, negative feedback and competitive binding are yet to be defined. Further investigation of the RTA404 pharmacology and molecular biology will provide new insights to drive the development of strategies to manipulate the balance among pathway responses under both physiological and disease conditions regulated by RTA404.

### 4.5. RTA404 Induced Intrinsic Apoptotic Pathway Initiated by Checkpoint Kinases in Malignant Glioma 

Leading to cell cycle arrest and inducing cell apoptosis are the two main strategies for suppressing cancerous tumors. As shown in Figure 7, RTA404 induces apoptosis and activates checkpoint kinases in MG. First, RTA404 activates checkpoint kinases. Upon treatment with RTA404, the relative intensities of CHK1, CHK2 and p-CHK2 were significantly upregulated in expression, and the intensity of p21 was significantly upregulated in expression also. It is implied that RTA404 activates the checkpoint kinases such as CHK1, CHK2 and p-CHK2, thereby causing cells to fail to cell progression, and ultimately leading to cell cycle arrest or inducing cell death. In addition, RTA404 induces cell apoptosis through reducing mitochondria membranes’ potential and activating the caspase 3 activities. Therefore, RTA404 activated the DNA checkpoint system and induced apoptosis through an intrinsic apoptotic pathway in established U87MG cells. 

## 5. Conclusions

Treatment with RTA404 reduces the viability of U87MG cells in both a time and dose-dependent manner. RTA404 treated cells had a significant increase in the percentage of apoptotic cells and in the of loss mitochondria membrane potential, in addition to a rise in the percentage of caspase-3 activity. Therefore, these results indicated that incubation with RTA404 inhibited cell viability and initiated cell apoptosis through an intrinsic apoptotic pathway. Although treatment with RTA404 led to cell cycle arrests in previous studies [19], without significantly change in mitosis index have found in RTA404 treatment. Based on the above results, it seems to imply that RTA404 activated the DNA checkpoint system and induced apoptosis through an intrinsic apoptotic pathway in established U87MG cells. Therefore, RTA404, a novel and potential anticancer drug, induces cell apoptosis and induces cycle arrest in GBM.

## Data Availability

The data used to support the findings of this study are available in this article’s Supplementary Materials.

## Supplementary Materials

The following are available online at https://www.mdpi.com/article/10.3390/jcm10214805/s1, All the data and statistical procedure in this experiment.

## Abbreviations

Antioxidant Response Element	ARE
2-cyano-3-,12-dioxoolean-1,9-dien-28-oic acid	CDDO
CDDO imidazolide	CDDO-Im
CDDO methyl ester	CDDO-Me
CDDOtrifluoroethyl amide	CDDO-TFEA
Glioblastoma multiforme	GBM
Malignant Glioma	MG
Kelch-like ECH-associated protein 1	Keap1
Nuclear factor erythroid 2-related factor 2	Nrf2
World Health Organization	WHO
